# High-Risk HPV Detection in Paraffin-Embedded Tissue from Cervical Lesions

**DOI:** 10.3390/ph17091201

**Published:** 2024-09-12

**Authors:** Micaela Almeida, Vitor Caeiro, Diana Costa, Lara Silva, Cíntia Sousa, Paula Pestana, Sofia Campelos, João Vale, Ana Cristina Ramalhinho, José Fonseca-Moutinho, Luiza Breitenfeld

**Affiliations:** 1 Health Sciences Research Centre (CICS), Faculty of Health Sciences, University of Beira Interior (UBI), Avenida Infante D. Henrique, 6200-506 Covilhã, Portugal; micaelacpalmeida@gmail.com (M.A.); vcaeiro8@gmail.com (V.C.); diana.raquel.costa@ubi.pt (D.C.); lara.silva@ubi.pt (L.S.); pgpestana@gmail.com (P.P.); jafmoutinho@fcsaude.ubi.pt (J.F.-M.); 2 Department of Pathology, Ipatimup Diagnostics, Rua Júlio Amaral de Carvalho, 45, 4200-135 Porto, Portugaljvale@ipatimup.pt (J.V.); 3 I3S—Instituto de Investigação e Inovação em Saúde, University of Porto, 4200-135 Porto, Portugal; 4 Clinical Academic Centre of Beiras (CACB), Edifício UBImedical, Estrada Municipal 506, 6200-284 Covilhã, Portugal; 5 Cova da Beira Local Health Unit, Alameda Pêro da Covilhã, 6200-251 Covilhã, Portugal

**Keywords:** hrHPV Detection kit, hrHPV genotyping, cervical cancer

## Abstract

Background: Human papillomavirus (HPV), a leading cause of cervical cancer, is present in most cases of the disease and ranks as the fourth most common cancer in women globally. Among the HPV types, fourteen (HPV 16/18/31/33/35/39/45/51/52/56/58/59/66/68) are recognized as high-risk (hrHPV), each with varying levels of oncogenic potential. Detecting and genotyping these hrHPV types in cervical lesions is crucial, requiring the development of new diagnostic methods. Methods: This study focuses on a retrospective analysis conducted on 44 women from the Cova da Beira Local Health Unit. We used the Anyplex™ II hrHPV Detection kit for hrHPV genotyping from paraffin-embedded cervical tissue samples. Results: hrHPV types were identified in 38 out of the 44 women. Genotyping revealed HPV-16 (55.3%), HPV-18/39/56/58/59 (5.3%), HPV-31 (21.1%), HPV-35 (7.9%), HPV-51/66 (2.6%), and HPV-52 (10.5%). Conclusions: This study demonstrates that the Anyplex™ II hrHPV Detection kit, originally designed for cervical cancer screening, is also effective for hrHPV genotyping in histological analyses. This methodology offers a simpler and more cost-effective approach for cervical cancer risk stratification. Its implementation in clinical practice could enhance the detection of hrHPV in cervical lesions, thereby contributing to more precise diagnoses and potentially more informed treatment strategies.

## 1. Introduction

Human papillomavirus (HPV) plays a crucial role in the development of cervical cancer, having been detected in 99.7% of all cases [1]. This high prevalence establishes HPV as the primary cause of cervical cancer and underscores the importance of targeted screening and prevention strategies. Globally, HPV is the fourth most common cause of cancer among women and is a leading cause of cancer-related mortality in 23 countries [2]. The International Agency for Research on Cancer (IARC) recognizes several HPV types as high-risk (hrHPV) due to their strong link with cervical cancer, including HPV-16/18/31/33/35/39/45/51/52/56/58/59, classified as Group 1 carcinogens. Additionally, HPV-68 and HPV-66 are categorized as probably carcinogenic (Group 2A) and possibly carcinogenic (Group 2B), respectively [3].

Screening programs employing both conventional and liquid-based cytology have contributed significantly to reducing cervical cancer incidence [4,5]. Nonetheless, hrHPV testing, particularly for hrHPV-16/18/31/33/35/39/45/51/52/56/58/59/66/68, has proven to be more sensitive than cytology in detecting high-grade intraepithelial lesions (HSILs). As a result, hrHPV testing is recommended as the primary screening tool by most scientific societies [6]. The World Health Organization (WHO) endorses a screen-and-treat approach, which includes a visual inspection with acetic acid (VIA) and hrHPV DNA tests that identify the 14 types of hrHPV [5].

In clinical practice, the risk of progressing to more severe conditions, such as cervical intraepithelial neoplasia grade 3 (CIN3+), a precursor to cervical cancer, is assessed through HPV testing and reflex cytology results. The current guidelines, including those released by the American Society for Colposcopy and Cervical Pathology (ASCCP), advocate for colposcopy when there is a 4% or greater probability of finding CIN3+ based on a combination of historical and current test results. This shift to risk-based decision making marks a significant advancement in cervical cancer screening and management, aiming to identify and treat high-grade lesions that have the potential to progress to invasive cancer [7,8].

For histological high-grade cervical lesions, validated tools to assess the risk of progression to cancer are still limited. A lower age, typically less than 25–30 years old, has often been used as a criterion to defer treatment [9]. Given that HPV types 16 and 18 are associated with more aggressive behavior and a higher risk of progression to invasive cancer [10], determining HPV types in histological lesions of HSIL could be valuable for risk stratification and treatment optimization.

In clinical practice, there is a growing need for simpler and more cost-effective techniques for HPV genotyping in histological lesions of HSIL. While hrHPV tests are commonly used to genotype samples collected from Pap tests, the identification of hrHPV genotypes in existing lesions is crucial, particularly when lesions have already progressed.

To our knowledge, this is the first time that the Anyplex™ II HPV HR Detection kit has been used for hrHPV detection in paraffin-embedded tissue from cervical lesions. This study aims to describe a protocol for DNA extraction from paraffin-embedded tissue from cervical lesions and subsequent hrHPV genotyping. This approach represents a novel application in the context of HR-HPV identification when the lesion has already occurred. This could potentially lead to improved prognosis and outcomes for patients. Additionally, our findings might support the integration of this approach into existing HPV screen-and-treat algorithms, potentially influencing future updates to HPV guidelines [8,9].

## 2. Results

In the present study, a total of 45 paraffin-embedded biopsy slides, each meticulously cut to a thickness of 10 μm and encapsulating HSILs, underwent genomic DNA extraction. Out of the initial 45 samples processed for genomic DNA extraction, the procedure was successfully completed in 44 samples. One sample proved to be challenging, and genomic DNA extraction was not possible. This may have been due to several possibilities like a lower amount of tissue, long-term storage, or handling error. Thus, the sample could not be considered. Therefore, the 44 successfully extracted genomic DNA samples were then genotyped for HPV using the Anyplex™ II HPV HR Detection kit, Catalog Nr. HP7E00X, (Seegene^®^, Seoul, Republic of Korea, acquired to Werfen, Carnaxide, Portugal).

Following the genotyping process, the collected data were analyzed using Seegene Viewer™ (Seegene^®^ Seoul, Republic of Korea, acquired to Werfen, Carnaxide, Portugal)).

Through this analysis, it was verified that the majority of the samples, namely 38 of 44 (equating to 86.4%), tested positive for one or more hrHPV types, and 6 samples (13.6%) tested negative for hrHPV types (Table 1).

In the analysis conducted in this study, the genotyping results from hrHPV detection reveal a diverse distribution of HPV types among the examined cases. In the data presented in Table 2, positivity for HPV-16 can be verified in 21 of the 38 HPV-positive samples (55.3%).

Furthermore, eight cases (21.1%) positive for HPV-31 were identified.

HPV-52 and HPV-35 were detected in four (10.5%) and three (7.9%) cases, respectively. The analysis also revealed lower frequencies of HPV-18, 39, 56, 58, and 59, with each of these types being found in two cases (5.3%). Lastly, the least common types detected in this cohort were HPV-51 and HPV-66, each found in only one case (2.6%).

We did not find any cases of HPV-33, HPV-45, or HPV-68 infection. This may have been due to the low estimated prevalence of these genotypes for the general female population of mainland Portugal aged 18 to 64 years (0.2%, 0.1%, and 0.2%, respectively) [11] and to the number of cases included in this study, which may have limited the number of findings.

In the present study, a significant finding was the identification of multiple hrHPV infections observed in nine cases (23.9%). The detailed data regarding multiple co-infections are clarified and summarized in Table 3. 

Among co-infections, double hrHPV infections were the most common, identified in eight cases. Specifically, HPV-16 was involved in three distinct combinations of double infections: two cases were a co-infection with HPV-16 and HPV-18, and one case was a combination of HPV-16 with HPV-35 and of HPV-16 with HPV-59. Other double co-infections verified were two cases positive for HPV-31 and HPV-39. Moreover, individual cases of HPV-35/58 and HPV-52/56 were found. 

Remarkably, a case of triple hrHPV infection was also found, which was positive for HPV-16, HPV-31, and HPV-35. 

These results indicate that the Anyplex™ II HPV HR Detection kit is feasible for genotyping cervical lesions. The use of the described protocol for DNA extraction from paraffin-embedded tissue from cervical lesions and subsequent hrHPV genotyping, which turned out, in our opinion, to be simple to execute, can be of great importance for the risk stratification of high-grade cervical lesions since the majority of the lesions were positive for hrHPV and some lesions presented multiple hrHPV infections. 

## 3. Discussion

Cervical cancer remains a significant global health challenge, with persistent infections by high-risk human papillomavirus (hrHPV) strains, notably HPV types 16 and 18, being identified as principal risk factors. This study emphasizes the critical role of the early detection and monitoring of hrHPV infections in facilitating early interventions and potentially preventing the progression to cervical cancer.

hrHPV types are detected in the vast majority of cervical cancer cases, contributing significantly to morbidity and mortality associated with the disease [3]. Our retrospective study analyzed 45 paraffin-embedded samples from cervical lesions, focusing on the genotyping of hrHPV. For this process, DNA extraction from paraffin-embedded slides was performed, allowing for hrHPV genotyping in such tissue samples using the Anyplex™ II HPV HR Detection kit. Thus, our study underscores the precision and effectiveness of the kit in detecting hrHPV in tissue from cervical lesions.

Screening programs, especially those incorporating hrHPV testing, play a crucial role in the prevention of cervical cancer. However, our findings highlight the importance of extending hrHPV genotyping capabilities to histological analyses of cervical lesions. This approach could enhance the accuracy of cervical cancer diagnoses and aid in the stratification of patient risk, thereby informing more tailored treatment strategies.

In the present work, in 44 of 45 cases (97.8%), it was possible to extract genomic DNA for multiplex real-time PCR amplification and hrHPV detection. Among these 44 cases, 86.4% of the lesions tested positive for at least one hrHPV type. HPV-16 was the most prevalent type (55.3%), which aligns with the global epidemiological data, as a leading cause of cervical cancer, highlighting its significant oncogenic potential and the importance of its early detection [12,13,14].

The second most prevalent hrHPV type was HPV-31; albeit it is less commonly implicated in cervical cancer when compared to HPV-16 and HPV-18, it is also classified as a high-risk oncogenic type [3]. The prevalence of HPV-31 is in accordance with that described in the literature for Europe [15]. 

After that, the most common HPV types were HPV-52 and 35 (10.5% and 7.9%, respectively). The prevalence of HPV-52 was similar to that found by Sousa et al., 2019 in a population of the northern region of Portugal [16]. However, HPV-35 was present in a higher number of cases in the present cohort when compared to the study in the northern region of Portugal, and it is closer to the prevalence of HPV-35 in Africa [15,16]. The cases studied revealed lower frequencies of HPV-18, 39, 56, 58, and 59, with each of these types being found in two cases. The less frequent types were HPV-51 and HPV-66, with each being found in only one case. The prevalence of these high-risk types is similar to that identified for Europe [15]. 

Despite the prevalence of each high-risk type, the data indicate the need for a more accurate approach taking into account the types of HPV. The methodology presented provides information on the types of HPV that lead to precancerous lesions, going beyond a screening program that detects the prevalence of infection. 

Moreover, we verified the multiple co-infections that underscore the complex interaction of the different HPV genotypes in the pathogenesis of cervical lesions and how they potentially impact the progression and management of the disease.

Almost a quarter of the lesions presented multiple hrHPV infections (23.8%). Similar results were found by Sousa et al. as 25.7% of the liquid-based cytology samples also had multiple infections [16]. hrHPV 16 was the more common type in co-infections, and there was one case of a triple co-infection (HPV-16/35/59), which illustrates the diversity of HPV interactions that can occur within the cervical epithelium, probably leading to a more heterogenic behavior in HSIL depending on the types of hrHPV present. This scenario highlights the potential for multiple high-risk HPV types to concurrently infect and influence the pathological landscape of cervical tissues. The presence of multiple hrHPV genotypes in a single case raises important questions about the interactions between different HPV types and their collective impact on the severity, progression, and treatment response of cervical lesions. 

This is the first time, to our knowledge, that paraffin-embedded slides from cervical lesions were genotyped using the Anyplex™ II HPV HR Detection kit, and this represents a significant methodological advancement, offering a more streamlined and efficient approach for earlier detection of the lesions and precursors of cervical cancer development. The kit used in the present work has so far been used for biopsies of liquid cytology specimens and now shows to also be an effective tool for paraffin-embedded samples from cervical lesions. 

Traditional methods for HPV detection often involve time-consuming and labor-intensive techniques. Using the referred kit for HPV detection in paraffin-embedded slides can provide a more efficient and accurate diagnostic method. This improved accuracy is vital in ensuring that individuals who are at risk receive appropriate follow-ups and treatments. The use of paraffin-embedded tissue slides is a common practice in pathology labs as they allow for the long-term storage of tissue samples. 

Detecting high-risk HPV in cervical lesions has clinical relevance, giving accurate information about whether the lesion is due to hrHPV and of which type. Therefore, it can aid clinicians in the identification of patients who may require closer monitoring and more accurate treatment.

This research contributes to the ongoing efforts in cervical cancer screening and prevention. By developing a more efficient and reliable method for detecting high-risk HPV, this study may have implications for public health programs and policies aimed at reducing the burden of cervical cancer, potentially reducing the need for unnecessary interventions and minimizing healthcare costs.

Ultimately, the clinical interest of this study lies in its potential to improve patient outcomes. The early detection of high-risk HPV can lead to timely interventions, which can significantly impact the prognosis and quality of life for individuals with pre-cancerous lesions.

In summary, this study on high-risk HPV detection using the Anyplex™ II HPV HR Detection kit in paraffin-embedded slides from cervical lesions contributes to the ongoing research in cervical cancer prevention and management. While it adds information to the existing body of knowledge, the potential impact of this study on diagnostic accuracy and clinical decision making should be viewed with cautious optimism. It represents an incremental step rather than a groundbreaking advancement in the field.

The findings of this study suggest possible applications in the stratification of HSIL risk and therapeutic management. However, it is important to recognize the inherent limitations to a study of this scale. The present work is a pilot study, and further laboratory studies with a larger sample size are necessary to validate the findings and to understand the full relevance of this protocol in a clinical setting. Additionally, clinical studies are required to determine the feasibility and utility of this technique, particularly in assessing the risk progression of cervical lesions.

In essence, while this investigation provides useful insights in the context of cervical cancer screening, it highlights the utility of hrHPV genotyping HSIL biopsies, which can be performed with the developed protocol using the Anyplex™ II HPV HR Detection kit.

## 4. Materials and Methods

### 4.1. Study Population

A retrospective study was performed using paraffin-embedded tissue samples from 45 women previously submitted to squamous high-grade cervical lesion excision in Cova da Beira Local Health Unit. Sample collection occurred at the Child and Women Department, Gynaecologic Oncology Division of Cova da Beira Local Health Unit, Covilhã, Portugal. This study was approved by the Ethics Committee of Beira Interior University with the code CE-UBI-Pj-2017-027.

### 4.2. DNA Extraction

The meticulous process of DNA extraction from tissue samples embedded in paraffin is a critical step for molecular analysis. 

Initially, prior to DNA extraction, 3 μm thick slides were stained with hematoxylin and eosin. The slides were assessed by two independent pathologists in order to confirm the presence of HSIL.

Upon confirmation of HSIL, DNA extraction was performed.

The process of preparing the slides for DNA extraction involves several meticulously executed steps to prevent cross contamination and ensure the purity of the DNA.

The first step in DNA extraction optimization was obtaining the correct thickness of the tissue; thus, 10 μm paraffin-embedded biopsy slides were obtained for each confirmed case of HSIL. 

Following the xylene treatment, the slides were submerged in xylene until the slide was completely covered. Incubation was carried out using a new sterile falcon tube of 50 mL for each slide in order to avoid cross contamination. This step is crucial for deparaffinization because paraffin, which embeds and preserves tissue, must be completely dissolved, allowing for the exposure of the underlying tissue and subsequent extraction of its DNA.

The slides were then individually transferred to a new 50 mL sterile falcon tube, and absolute ethanol was used until the slide was completely submerged. The incubation period was 5 min at room temperature. After that, the slides were allowed to dry at room temperature for 5 to 10 min. 

The next step involved the physical retrieval of the tissue from the slide. A new sterile scalpel was used for each sample; the tissue was carefully scraped off the slide into a 1.5 mL microtube. This is a delicate process requiring precision to ensure that the tissue is successfully collected without contamination. No flow should be present in order to avoid the escape of the scraped tissue. 

The extraction of genomic DNA was performed using the QIAamp DNA FFPE Tissue Kit (Qiagen, Germantown, MD, USA) according to the manufacturer’s instructions. The protocol provided by the manufacturer was followed meticulously, and filtered pipette tips were used. The entire process of DNA extraction was performed in a dedicated workstation in order to maintain a contamination-free environment.

After extraction, the samples were stored at −20 °C.

### 4.3. HPV Genotyping

For hrHPV genotyping, the Anyplex™ II HPV HR Detection kit, Catalog Nr. HP7E00X, (Seegene^®^, Seoul, Republic of Korea, acquired to Werfen, Portugal) was used following the manufacturer’s instructions [17]. A multiplex real-time PCR (CFX96 PCR from Bio-Rad, Hercules, CA, USA) was performed. The kit enables the simultaneous genotyping of the 14 HPV types, including 12 high-risk types identified as Group 1 carcinogens (HPV-16/18/31/33/35/39/45/51/52/56/58/59). Additionally, it encompasses HPV-66, classified under Group 2B as possibly carcinogenic, and HPV-68, classified under Group 2A as probably carcinogenic [3,17]. The Anyplex™ II HPV HR Detection kit provides an internal control for each sample and positive and negative controls for each plate for the real-time PCR reaction. The meticulous control system is pivotal for ensuring the accuracy and reliability of the PCR results, offering an added layer of validation to the genotyping process. By including these controls, the kit effectively minimizes the potential for false positive or false negative results, thus providing a higher degree of confidence.

To prevent cross contamination, the PCR mixture was prepared within a UV PCR cabinet. Following this step, the addition of DNA was carried out in a vertical laminar flow cabinet. This cabinet ensures a sterile airflow, safeguarding the integrity of the samples and the accuracy of the genotyping process. 

For genotyping, filtered pipette tips were also used, also as dedicated pipettes, to avoid cross contamination. 

PCR data were analyzed using Seegene Viewer™ (Seegene^®^) software, Version 3, which is specifically designed to interpret data generated by multiplex real-time PCR. The internal controls of the kit and the algorithm of the software allowed us to confirm the DNA amplification and the hrHPV type(s) present in each HSIL.

## Figures and Tables

**Table 1:** The HPV statuses of the 44 samples included in this study.

HPV Status	*n* (%)
	44 (100)
HPV-positive	38 (86.4)
HPV-negative	6 (13.6)

**Table 2:** hrHPV genotyping.

HPV Genotypes	*n* (%)
HPV-16	21 (55.3)
HPV-18	2 (5.3)
HPV-31	8 (21.1)
HPV-33	0
HPV-35	3 (7.9)
HPV-39	2 (5.3)
HPV-45	0
HPV-51	1 (2.6)
HPV-52	4 (10.5)
HPV-56	2 (5.3)
HPV-58	2 (5.3)
HPV-59	2 (5.3)
HPV-66	1 (2.6)
HPV-68	0

**Table 3:** Co-infections of HPV genotypes.

Co-Infection	*n*
HPV-16 and HPV-18	2
HPV-16 and HPV-35	1
HPV-16 and HPV-59	1
HPV-16, HPV-31 and HPV-35	1
HPV-31 and HPV-39	2
HPV-35 and HPV-58	1
HPV-52 and HPV-56	1

## Data Availability

Data is contained within the article.
